# Relationships between protein degradation, cellular senescence, and organismal aging

**DOI:** 10.1093/jb/mvae016

**Published:** 2024-02-13

**Authors:** Jun Hamazaki, Shigeo Murata

**Affiliations:** Laboratory of Protein Metabolism, Graduate School of Pharmaceutical Sciences, the University of Tokyo, 7-3-1, Hongo, Bunkyo-ku, Tokyo 1130033, Japan; Laboratory of Protein Metabolism, Graduate School of Pharmaceutical Sciences, the University of Tokyo, 7-3-1, Hongo, Bunkyo-ku, Tokyo 1130033, Japan

**Keywords:** aging, autophagy, proteasome, proteostasis, senescence

## Abstract

Aging is a major risk factor for many diseases. Recent studies have shown that age-related disruption of proteostasis leads to the accumulation of abnormal proteins and that dysfunction of the two major intracellular proteolytic pathways, the ubiquitin-proteasome pathway, and the autophagy-lysosome pathway, is largely responsible for this process. Conversely, it has been shown that activation of these proteolytic pathways may contribute to lifespan extension and suppression of pathological conditions, making it a promising intervention for anti-aging. This review provides an overview of the important role of intracellular protein degradation in aging and summarizes how the disruption of proteostasis is involved in age-related diseases.

## Graphical Abstract

**Graphical Abstract:**
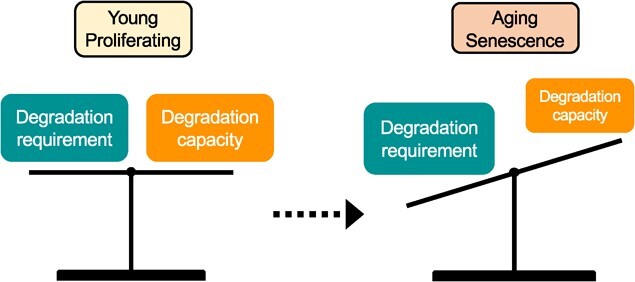

Aging is a major risk factor for various diseases, including cancer, cardiovascular diseases, and neurodegenerative diseases. Maintaining proteome balance in response to various stresses becomes challenging with age, resulting in a decline in proteostasis network capacity. Consequently, an outstanding feature shared by many age-associated diseases is the disruption of proteostasis, which leads to the accumulation of protein aggregates. Recent studies on proteomic changes associated with aging provide insights for improving proteostasis and developing anti-aging strategies *(**1**)*. For instance, studies in aged mice have shown increased immunoglobulins and immunoproteasomes in many tissues, indicating widespread age-linked inflammatory responses *(**2**)*. However, our understanding of changes shared in different organs during aging is still limited, and the underlying pathways and factors involved in senescence remain enigmatic *(**3**)*.

Proteomic changes in aging primarily depend on the regulation of protein levels rather than gene expression through transcription. Key factors include decreased activity of protein degradation and changes in ribosomal occupancy *(**4**)*. Protein synthesis and degradation are believed to decline with age, although there is significant variability among organs and tissues. Proteomic analysis in *Caenorhabditis elegans* has shown a reduction of approximately 40% in proteome turnover with age, while the antioxidant system and the ubiquitin-proteasome system remain relatively constant *(**5**)*. In contrast, analysis in mice showed that there are few proteins whose abundance increased more than 2-fold in aged mice compared to young mice, implying that the global proteomic changes with aging are slight *(**6**)*. Analyses in rats confirmed that proteomic changes during aging are minor and vary from organ to organ and also showed that aging caused changes in translation efficiency, phosphorylation state and subcellular localization of proteins and composition of protein complexes *(**7**)*.

Studies in mice have shown that protein lifetimes were extended by 20% in aged brains compared to young brains. Specifically, the lifetime of neuroprotective proteins was extended, while that of some mitochondrial proteins was shortened. Furthermore, the turnover of proteins that require substantial energy investments for biosynthesis, such as long-chain polypeptides, decreased, suggesting a significant interplay between energy cost and protein homeostasis in the aging process *(**8**)*.

Activation of the proteasome and autophagy has been shown to extend the lifespan of model organisms such as fruit flies and mice. This suggests an inverse correlation between protein turnover rates and organismal lifespan *(**9**)*. This evidence supports the connection between age-related proteomic changes and protein degradation across species.

## Protein Synthesis during Aging

Accuracy of translation is also believed to play a significant role in lifespan. Approximately 30% of newly synthesized proteins are structurally defective and are degraded during or immediately after translation, and under certain circumstances, the percentage can rise to nearly 60% *(**10**)*. Fibroblasts derived from naked mole rats, a long-lived rodent with a lifespan of more than 20 years, have higher translation accuracy and produce fewer aberrant proteins than mouse fibroblasts *(**11**)*. A decline in translation accuracy is associated with aging and disease development, and drugs such as rapamycin and the ribosomal 40S subunit RPS23 mutation have been shown to extend lifespan by improving translation accuracy *(**12**)*.

Studies in nematodes and yeast have shown that translation efficiency in base-rich regions decreased with age, while ribosomal arrest and collisions increased. Exacerbated proteostasis due to increased aggregation of nascent polypeptides with age has been demonstrated, suggesting an increased burden of ribosome quality control with age *(**13**)*.

The integrated stress response (ISR) is a stress signaling pathway that regulates translation via phosphorylation of the translation initiation factor eIF2. Although its relevance to aging remains unclear, inhibition of the ISR has been shown to extend the lifespan in *C. elegans* without a global decrease in protein synthesis *(**14**)*.

## Cellular Senescence and Protein Degradation

**Fig. 1:**
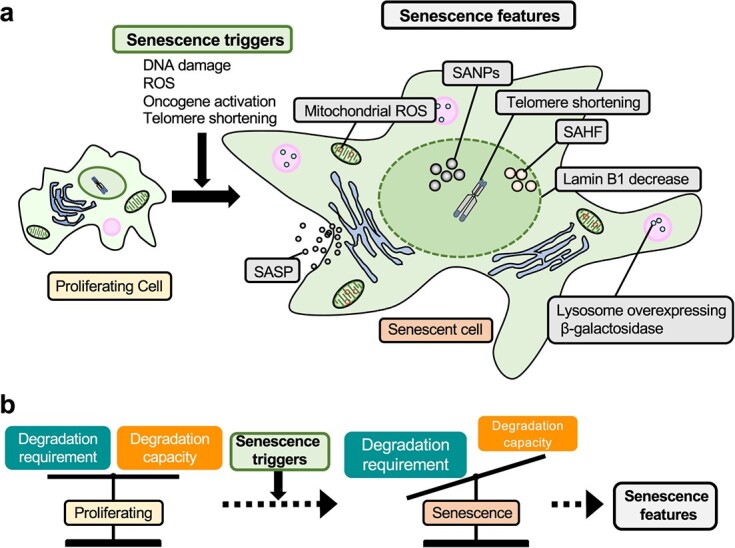
**Protein degradation and Cellular senescence. a.** Proliferating cells undergo cellular senescence accompanied by the cessation of cell division due to various stresses known as senescence triggers. **b.** Recent advances in cellular senescence research have revealed that the modulation of protein degradation in senescent cells controls the properties and characteristic features of senescent cells.

Repeated passaging of normal human somatic cells leads to mitotic arrest mainly due to telomere shortening. Cellular senescence is the state in which cells irreversibly stop proliferating due to various stresses, such as DNA damage, replication stress, mitochondrial dysfunction, reactive oxygen species (ROS) production and oncogene induction (Fig. 1a).

Senescent cells have many characteristics. The most widely used biomarker to detect senescent cells is the accumulation of a lysosomal enzyme called ‘senescence-associated β-galactosidase (SA-β-gal)’. Senescent cells also show an abnormally enlarged morphology with enlarged nuclei and foci of condensed chromatin, termed senescence-associated heterochromatin foci (SAHF). Mitochondria increase with altered membrane potential, ROS production and oxygen consumption in senescent cells. Senescent cells develop a senescence-associated secretory phenotype (SASP), in which they secrete various inflammatory proteins that induce inflammation in the surrounding tissues. Additionally, senescent cells are metabolically active, despite not proliferating, and exhibit ATP levels comparable to those of proliferating cells *(**15**)*. Cellular senescence is believed to act as a mechanism for suppressing tumors, but it has also been shown to be associated with increased tumor growth through SASP. Although the contribution of cellular senescence to aging is considered unquestionable, its physiological role remains to be fully elucidated. Recent advances in cellular senescence research have revealed the importance of proteolysis, especially the ubiquitin-proteasome system (UPS) and the autophagy-lysosome pathway in the proteostasis of aging cells *(**16**)*. Under proliferative conditions, the UPS and the autophagy-lysosomal pathways, operating independently or cooperatively, maintain the proteome. However, under chronic stress, such as senescence triggers, the degradation capacities become insufficient compared to the degradation requirements to maintain the proteome. This imbalance is exacerbated by the accumulation of damaged proteins, leading to a vicious cycle that results in further stress and perturbation of proteostasis, resulting in senescence with multiple features (Fig. 1b).

## The Ubiquitin System during Aging

**Fig. 2:**
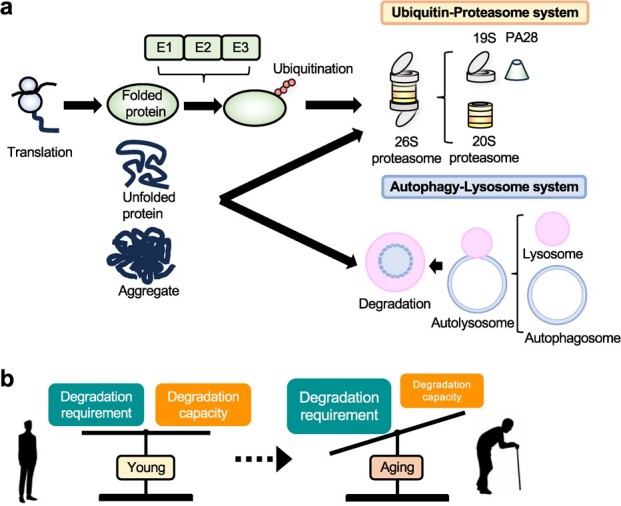
**Protein degradation during aging. a.** After translation, the functional folded protein undergoes ubiquitination at an appropriate time and is primarily degraded by the 26S proteasome. Some proteins are known to be degraded exclusively by the autophagy-lysosome pathway. While proteasomes are also involved in the degradation of unfolded proteins, they are not efficient at degrading aggregated proteins. Aggregated proteins are believed to be degraded and removed mainly by the autophagy-lysosome pathway. **b.** In young individuals, the supply and demand for intracellular protein degradation are balanced. During the aging process, this balance within cells becomes disrupted, resulting in the abnormal accumulation of proteins and a subsequent decline in cellular function.

UPS is responsible for degrading short-lived and misfolded proteins in eukaryotes (Fig. 2a). Proteins tagged with polyubiquitin chains that carry degradation signals, typically K48 chains, are recognized and degraded by the 26S proteasome. UPS plays a pivotal role in proteome formation, being involved in the degradation of 70% to 90% of intracellular proteins *(**17**)*.

Proteomic analysis of ubiquitinated proteins in *C. elegans* was conducted to investigate the relationship between aging and ubiquitinated proteins. Contrary to expectations, the results showed a global decrease in ubiquitinated proteins with aging, suggesting an increase in deubiquitinating enzyme activity that decreases the accumulation of ubiquitinated proteins without degradation *(**18**)*. In contrast, the state of protein ubiquitination remained constant or increased in aged individuals under lifespan-extending conditions, such as dietary restriction and reduced insulin signaling. Indeed, it has been demonstrated that certain proteins subject to proteasomal degradation, such as the intermediate filament IFB2 and EPS8 of the RAC signaling system, accumulated in a non-ubiquitinated state during aging and affected lifespan *(**18**)*. These findings highlight the significant impact of age-related changes in degradation targets on lifespan.

Proteomic investigations into ubiquitinated proteins in *Drosophila melanogaster* provided further insights*.* An age-associated increase in the ubiquitination of the long-lived protein histone 2A was observed. This phenomenon was subsequently validated in mice and humans. Intriguingly, a mutant fly with reduced levels of ubiquitinated H2A showed an extended lifespan, suggesting a role for ubiquitination in age-related epigenetic changes *(**19**)*.

The human genome encodes more than 600 ubiquitin ligases, each determining the substrate selectivity of the UPS machinery *(**20**)*. Each ubiquitin ligase functions to ubiquitinate its targets in different cells and physiological events. Several ubiquitin ligases have been reported to reduce the accumulation of aggregating proteins.

LONRF2 is a ubiquitin ligase that is specifically expressed in neurons. It has been shown to be induced in senescent cells and to ubiquitinate aggregate-prone proteins, such as TDP-43 and hnRNP M1. Mice lacking *Lonrf2* showed age-dependent exacerbation of neurodegenerative pathologies and shortened lifespan. Conversely, increasing LONRF2 expression ameliorated neurite shortening in motor neurons of amyotrophic lateral sclerosis patients *(**21**)*.

TRIM11 is a member of the TRIM family of proteins that are unique to metazoans. A reduction in TRIM11 protein levels has been reported in Alzheimer's disease (AD) brains, and TRIM11 has been suggested to play a role in the SUMOylation of mutant tau as well as excess normal tau and its subsequent degradation by proteasomes. TRIM11 has also been shown to have two additional functions: as a molecular chaperone that prevents tau misfolding and as a disaggregase that dissolves tau fibrils, although the contribution of SUMOylation activities to these functions has not been defined. Overexpression of TRIM11 alleviated progressive neurodegeneration and improved motor function *(**22**)*.

## The Proteasome and Cellular Senescence

The 26S proteasome is multi-catalytic protease complex responsible for the degradation of ubiquitinated proteins. It is formed by the association of 19S regulatory particles (RP) at both ends of the barrel-shaped 20S core particle (CP) with protease activity. The RP has functions, such as recognizing ubiquitin chains, deubiquitination and protein unfolding *(**23**)*.

Proteasome activity decreases during aging, and modest proteasome inhibition can induce cellular senescence *(**24**,**25**)*. Moreover, cells overexpressing the 20S CP subunit β5 have been shown to exhibit increased proteasome activity, acquire resistance to proteasome inhibitors and delay cellular senescence *(**26**)*. However, the dynamics and role of the proteasome in cells that have completed senescence remained unclear. In senescent cells, the expression of proteasomal genes decreased, resulting in a reduction of 26S proteasomes. Intriguingly, it was further unveiled that the senescent cells formed liquid droplets that contained the 26S proteasome. These foci were distinct from other known nuclear foci and named ‘senescence-associated nuclear proteasome foci (SANPs)’. SANPs formation required ubiquitinated proteins and RAD23B, a shuttle factor that delivers ubiquitinated proteins to the proteasome, like previously known stress-induced nuclear proteasome foci *(**27**)*. However, unlike the previous proteasome foci, proteasome activity was also required for SANPs formation. Inhibiting the formation of SANPs enhanced mitochondrial activity, suggesting the pivotal role of SANPs in regulating mitochondrial function in senescent cells, possibly by degrading some nuclear proteins in senescent cells *(**28**)*.

PD-1 is an immune checkpoint receptor expressed on the surface of antigen-stimulated T cells. The PD-1 ligand PD-L1 is known to be expressed on antigen-presenting cells and cancer cells, and the interaction of these proteins contributes to the immune escape of cancer cells. Some senescent cells express PD-L1 to evade the immune system. PD-L1^+^ senescent cells show lower proteasome activity compared to PD-L1^−^ senescent cells, and treatment of senescent cells with proteasome inhibitors increased the population of PD-L1 expressing cells. It is known that Nrf1 is a transcription factor that orchestrates the compensatory induction of proteasome genes in response to proteasome impairment *(**29**)*. Enhanced proteasome expression through overexpression of constitutively active mutant Nrf1 decreased the population of PD-L1^+^ senescent cells, suggesting that protein degradation by the proteasome regulates senescent cell survival via regulation of PD-L1 stability *(**30**)*.

Proteasome dysfunction is not always a contributing factor to neurodegeneration. Cysteine string protein α (CSPα) is a presynaptic chaperone protein responsible for SNARE maintenance in the presynaptic terminal, and the loss of CSPα causes synapse degeneration due to the reduction of dysfunctional SNARE proteins through degradation by the proteasome. In the case of neurodegeneration that develops in CSPα-deficient mice, proteasome inhibition actually suppressed neurodegeneration by ameliorating deficiency in SNARE complex formation, resulting in a lifespan-extending effect. This suggests that for some neurodegenerative diseases, a temporary decrease in proteasome activity may be beneficial to the cells, depending on the cause of neurodegeneration *(**31**)*.

## Proteasome Activation as a Promising Anti-Aging Strategy

Proteomic analysis of the African killifish brain has identified a decrease in proteasome activity as an early sign of aging, in addition to decreased stoichiometry and increased protein aggregation of protein complexes such as ribosomes and proteasomes in the aged brain. Furthermore, correlation analysis between gene expression in fin biopsies at a young age and the lifespan of each individual in the African killifish showed that the group with the greatest age-dependent down-regulation of proteasome expression at a young age had a shorter lifespan compared to the groups with up-regulation of proteasome expression *(**32**)*.

In contrast to mice, where the level of ubiquitinated proteins increases with age, the level of ubiquitinated proteins does not change in naked mole rats *(**33**)*. The liver of the naked mole rat has higher proteasome activity, lower levels of ubiquitinated proteins, and resistance to proteasome inhibitors compared to mice *(**34**)*. Furthermore, the study found that both the proteasome and autophagy were more strongly induced in naked mole rat fibroblasts than in mouse fibroblasts *(**35**)*.

Several studies have reported that enhancing proteasome function can reduce age-related phenotypes. Germ cell ablation in *C. elegans* is known to extend the lifespan. In this model, the FOXO transcription factor DAF-16 induced upregulation of the 19S RP subunit Rpn6, in turn, increased proteasome activity in somatic cells. *Caenorhabditis elegans* overexpressing Rpn6 exhibited increased proteasome function, tolerance to proteotoxic stress and prolonged lifespan. These findings suggest that enhancing proteasome function may be an anti-aging strategy *(**36**)*. In *D. melanogaster*, old flies have reduced 26S proteasomes and are more sensitive to proteasome inhibitors in terms of lifespan *(**37**)*. The overexpression of the 19S RP subunit Rpn11 enhanced proteasome function and suppressed neurodegenerative pathology caused by the accumulation of ubiquitinated proteins and polyQ protein accumulation in the compound eye *(**38**)*. Mice with systemic overexpression of Nrf1 exhibited increased proteasome synthesis. When crossed with a mouse model of human blindness, they showed a delayed age-related loss of photoreceptor neurons *(**39**)*.

Proteasome dysfunction has been observed in the brains of ad patients. Preventing proteasome dysfunction has been shown to reduce mortality rates and cognitive decline in fly models of AD. Remarkably, these benefits extend to mice with neuron-specific overexpression of the 20S CP subunit β5 in the AD mouse model. Furthermore, a newly developed peptide that allosterically enhances proteasome activity inhibited cell death and cognitive decline in fly and mouse models of AD *(**40**)*.

An activator complex called PA28 is also known to associate with 20S CP instead of 19S RP. Metazoans have the prototype PA28γ, while vertebrates have additional IFN-γ-inducible PA28α and PA28β. Lowering body temperature is an important mechanism for extending lifespan in both poikilotherms and homeotherms. In *C. elegans,* PA28γ (PSME-3) selectively enhanced the trypsin-like activity of the proteasome under low-temperature conditions (15°C). Induction of PA28γ expression by TRPA1 channel and the transcription factor NHR49 at low temperature was required for the extension of lifespan, inhibition of age-related reduction in proteolysis, and suppression of protein aggregation in age-related disease models, such as Huntington's disease, while PA28γ had no effect on lifespan at normal temperature. PA28γ-mediated proteasome activation and aggregation inhibition were also observed in human cells under mild low-temperature conditions (36 °C), indicating that the association between proteasomes and lifespan at low temperatures is evolutionarily conserved *(**41**)*.

As discussed above, many examples have shown that increasing proteasome activity generally extends lifespan and inhibits the development of age-related diseases, indicating that enhancing proteasome function is a promising anti-aging strategy.

## The Autophagy-Lysosome System during Aging

The autophagy-lysosome system is responsible for the large-scale degradation of intracellular components encompassing proteins, lipids, nucleic acids and organelles *(**42**)* (Fig. 2a). A growing body of evidence showed that autophagy activity declines with age in various organisms, suggesting that the decline of autophagy is a fundamental feature of aging. Increased lysosomes, as measured by SA-β-gal activity, are a hallmark of senescent cells, and it has been reported that this increase is due to lysosomal dysfunction in senescent cells. Lysosomes in senescent cells have a high pH, increased membrane damage and decreased protein degradation. TFEB/TFE3, a master regulator of lysomal biogenesis and autophagy, was shown to increase compensatory lysosomal biogenesis *(**43**)*.

Lifespan extension in *C. elegans* was achieved by inducing the HLH-30 transcription factor, an orthologue of TFEB, which regulates genes related to lysosome and autophagy, or the selective autophagy receptor p62/SQSTM-1. Similarly, overexpression of the Atg genes in flies (Atg1 and Atg8a) and mice (Atg5) also extended lifespan *(**44**)*. Conversely, Rubicon, an autophagy suppressor, increased in an age-dependent manner in nematodes, flies and mice and was shown to suppress autophagy in aged organisms. In contrast, Rubicon is downregulated in long-lived nematodes and calorie-restricted mice. Knockdown of Rubicon in nerves prolonged lifespan and ameliorated aging phenotypes in nematodes and flies. This collective evidence suggests that suppression of autophagy by Rubicon is a hallmark of aging *(**45**)*. However, Rubicon expression was decreased in aged adipose tissue, and increased autophagy exacerbated metabolic disorders by excessive degradation of the PPARγ coactivators SREC-1 and TIF2 *(**46**)*.

The transcription factor MondoA has been found to regulate cellular senescence through two pathways: activation of autophagy by suppressing Rubicon and maintenance of mitochondrial function by the peroxiredoxin Prdx3 *(**47**)*. Furthermore, the nematode orthologue MML1 enhanced autophagy in peripheral tissues and suppressed oxidative stress through the peroxidase MLT7 by inducing the glutamate transporter GLT-5 in nerves, thereby contributing to lifespan extension in germline-deficient nematodes *(**48**)*.

In *D. melanogaster*, short-term administration of the mTOR inhibitor rapamycin in early adulthood has been shown to extend lifespan. This was due to the short-term enhancement of autophagy in the intestine, which persisted even after rapamycin withdrawal. Furthermore, short-term rapamycin treatment in mice has been observed to improve intestinal function *(**49**)*.

## The Autophagy-Lysosome System and Cellular Senescence

There have been conflicting results regarding the changes and significance of autophagy activity in cellular senescence, and the mechanism and significance of autophagy activity remain unclear *(**44**)*. Autophagy is known to be transiently activated during senescence induction, followed by feedback through the PI3K-mTOR pathway. The overexpression of the autophagy factor ULK3 induced cellular senescence along with autophagy, while inhibition of autophagy suppressed the senescence phenotype, suggesting that autophagy may promote rapid changes in cellular metabolism during the senescence induction process *(**50**)*.

It has been demonstrated that APOE expression increased with age in human mesenchymal progenitor cells and that overexpression of APOE induced cellular senescence. Autophagy is known to be responsible for the decay of nuclear lamin B1, a hallmark of senescent cells *(**51**)*. It has been suggested that nuclear-localized APOE also acts as an epigenetic destabilizing factor that contributes to disruption of nuclear membrane proteins and heterochromatin via autophagy, promoting senescence *(**52**)*.

Furthermore, autophagy deficiency in AD mouse models can lead to the development of senescence-associated microglia characterized by features, including cell growth arrest, increased Cdkn1a/p21^Cip1^, dystrophic forms and SASP. This suggests that autophagy plays a crucial role in microglia in maintaining homeostasis and suppressing senescence *(**53**)*.

## Senolytic Strategy and Autophagy-Lysosome

The accumulation of senescent cells can cause inflammation and tumor promotion. Therefore, the specific elimination of senescent cells, known as senolysis, is being considered as a new anti-aging strategy. ARV825, an inhibitor of the BET family protein BRD4, has been found to have senolytic effects. ARV825 suppressed the development of liver cancer in obese mice by eliminating senescent hepatic stellate cells through the suppression of DNA damage repair and the induction of autophagy gene expression *(**54**)*.

A senolytic approach targeting lysosomal abnormalities associated with aging was reported. Senescent cells synthesize a large amount of proteins, which can lead to the accumulation of aggregated proteins in lysosomes. This accumulation can cause lysosomal damage and lower intracellular pH. GLS1, a kidney-type glutaminase, was expressed to promote the hydrolysis of glutamine and induce ammonia production, which helped to neutralize the intracellular pH. The GLS1 inhibitor BPTES has also been shown to effectively remove senescent cells in both cell culture and mouse organs *(**55**)*.

## Conclusion and Perspectives

This review underscores the crucial role of proteolysis in aging, revealing how proteostasis disruption contributes to age-related diseases (Fig. 2b). Research across various model organisms has highlighted the heterogeneity in aging-associated proteome changes, emphasizing the complexity of aging at the molecular level. Future research should focus on unraveling the intricate mechanisms of proteostasis in aging. Modulating proteolysis pathways presents promising avenues for anti-aging interventions. Investigating the dual role of cellular senescence in tumor suppression and promotion is critical for developing effective senolytic or senostatic therapies.

In summary, the field of aging and proteolysis presents vast opportunities for discovery and therapeutic advancements. Continued research in this area is crucial for improving our understanding of the aging process and developing interventions to enhance healthy lifespan in aging populations.
